# Forebrain control of breathing: Anatomy and potential functions

**DOI:** 10.3389/fneur.2022.1041887

**Published:** 2022-11-01

**Authors:** Karl M. Schottelkotte, Steven A. Crone

**Affiliations:** ^1^Department of Pharmacology and Systems Physiology, University of Cincinnati College of Medicine, Cincinnati, OH, United States; ^2^Division of Pediatric Neurosurgery, Cincinnati Children's Hospital Medical Center, Cincinnati, OH, United States; ^3^Division of Developmental Biology, Cincinnati Children's Hospital Medical Center, Cincinnati, OH, United States; ^4^Department of Neurosurgery, University of Cincinnati College of Medicine, Cincinnati, OH, United States

**Keywords:** forebrain, control of breathing, limbic system, cortex, respiratory circuits

## Abstract

The forebrain plays important roles in many critical functions, including the control of breathing. We propose that the forebrain is important for ensuring that breathing matches current and anticipated behavioral, emotional, and physiological needs. This review will summarize anatomical and functional evidence implicating forebrain regions in the control of breathing. These regions include the cerebral cortex, extended amygdala, hippocampus, hypothalamus, and thalamus. We will also point out areas where additional research is needed to better understand the specific roles of forebrain regions in the control of breathing.

## Introduction

Breathing is an essential function for humans during every waking and sleeping moment to provide movement of air in and out of the lungs (1–3). Our oxygen demands are dynamic and constantly changing depending on our activity level, emotional state, health status, and current behaviors. Because of this, the brain must constantly ensure that our breathing is appropriately matched to our physiological state and behavior. For example, our breathing rate and/or volume changes in anticipation of altered needs for gas exchange and tissue oxygenation during exercise or other physical activities (4). This feed-forward control is critical for maintaining homeostasis because there is no known mechanism for sensing gas exchange in the muscle or lungs. Breathing changes with emotional states as well, as the feelings of stress and fear can cause hyperventilation (5–7). Although the basic pattern of respiration (inspiration, post-inspiration, and expiration phases) is generated by neurons in the brainstem and transmitted to respiratory muscles via spinal circuits (1, 2, 8), these neurons are influenced by other brain regions in order to ensure that breathing is appropriate for the current situation.

Breathing, unlike other autonomic processes such as heart rate and blood pressure, can be modulated voluntarily in addition to autonomically (9–11). For example, singers and musicians that play wind instruments need precise control over their breathing to produce the correct notes and tones. Mindfulness exercises such as meditation and yoga utilize deliberate and precise breathing methods to elicit calming responses from the body, including lowering blood pressure and heart rate. Competitive weightlifters are among a variety of athletes that use methodic breathing techniques such as hyperventilating before their lift to provide sympathetic activation to maximize strength during their lift. Swimmers pace their breathing to ensure that they do not inadvertently inhale water. Thus, intentionally pacing respiration or modulating breathing volume is a tool that humans and animals use to control their own physiology.

**Figure 1:**
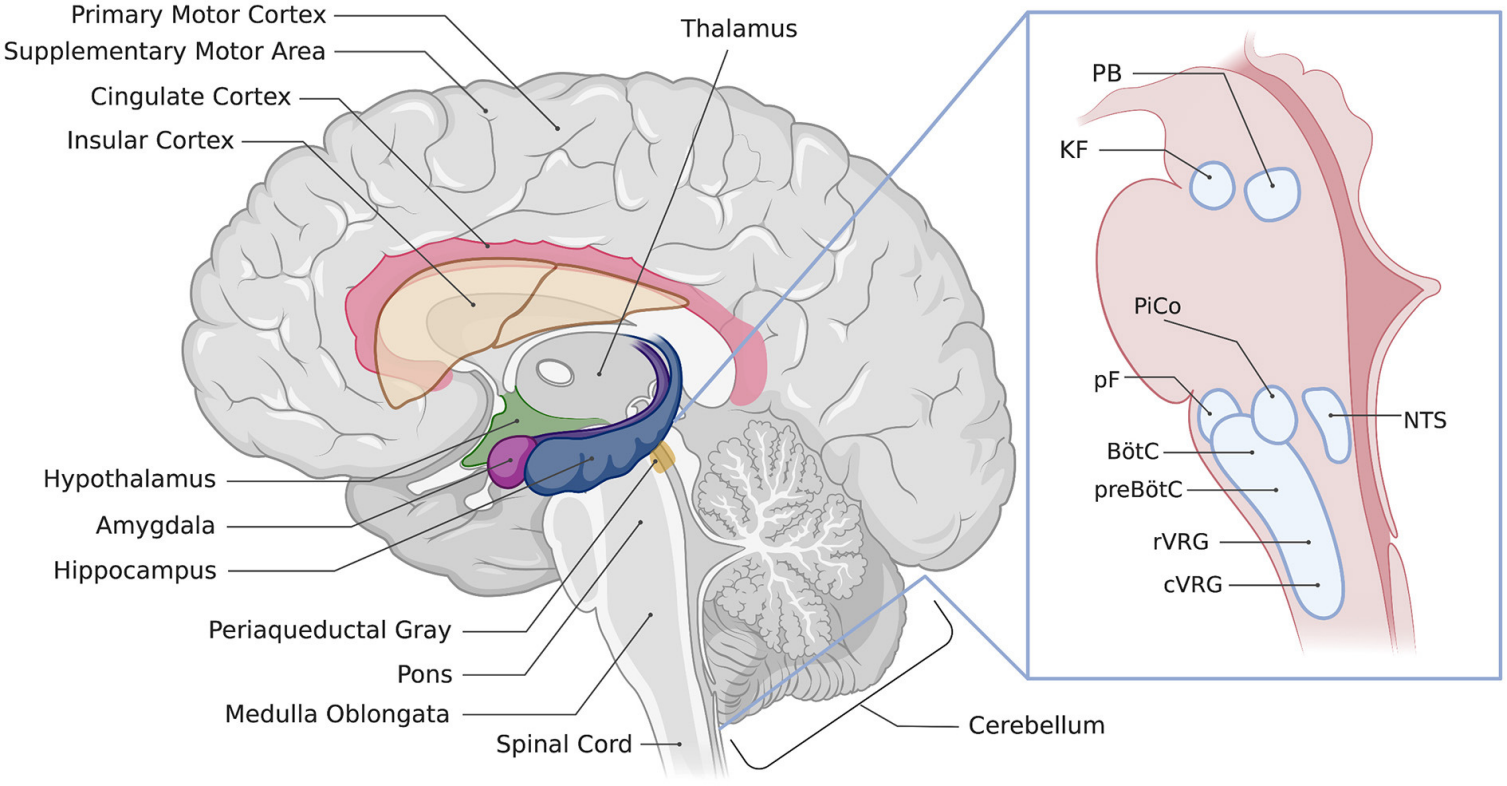
Location of regions involved in the control of breathing. Illustration of a sagittal section of the human brain showing the approximate location of respiratory-associated brain regions discussed in the text. The inset image shows brainstem respiratory regions, PAG, periaqueductal gray; PB, parabrachial nuclei; KF, Kölliker-Fuse nucleus; PiCo, post-inspiratory complex; pF, parafacial respiratory groups; NTS, nucleus of the solitary tract, BötC, Bötzinger complex; preBötC, preBötzinger complex; rVRG, rostral ventral respiratory group; cVRG, caudal ventral respiratory group. Created with BioRender.com.

The forebrain is important for the planning and execution of movements, sensory processing, regulating sleep wake states and behavioral responses to emotions such as stress and fear (5, 7). Each of these functions can have an impact on breathing. For example, fear is linked with a variety of respiratory changes- you may gasp if you are startled, you might find yourself holding your breath when scared, or even hyperventilating as your body prepares its fight or flight response. Sleep/wake states strongly influence the control of breathing, with important consequences if this relationship is dysfunctional, such as sleep apnea, congenital central hypoventilation syndrome, sudden infant death syndrome, or sudden unexpected death in epilepsy. Thus, we propose that the forebrain may be important for ensuring that breathing matches current and anticipated emotional, behavioral, and physiological needs. However, the circuits and mechanisms by which the forebrain exerts control over breathing are only partly understood. This review will summarize anatomical and functional evidence implicating forebrain regions in the control of breathing. These regions include the cerebral cortex, extended amygdala, hippocampus, hypothalamus, and thalamus (Figure 1). We will also point out areas where additional research is needed to better understand the specific roles of forebrain regions in the control of breathing.

## Brainstem and midbrain control of breathing

The brainstem is critical for the generation of respiratory rhythm, patterning of motor output, and adapting respiration to changes in blood gasses to ensure adequate ventilation at all times (1–3, 8, 12). Historically, the brainstem has been broadly divided into the ventral respiratory column, dorsal respiratory group, the parafacial respiratory group, and the pontine respiratory group. Here, we describe the main structures within these groups responsible for the control of breathing to provide a foundation for understanding how forebrain structures might modify breathing via their connections to these structures.

### Ventral respiratory column and the triple oscillator model

The pre-Bötzinger complex (preBötC), post-inspiratory complex (PiCo), and lateral portion of the parafacial respiratory group (pF_L_) are thought to be oscillatory rhythm generators responsible for driving the inspiratory, post-inspiratory, and expiratory phases of breathing, respectively (13). The preBötC is part of the ventral respiratory column that also includes the Bötzinger complex (BötC), rostral ventral respiratory group (rVRG), and caudal ventral respiratory group (cVRG) (1). The preBötC drives inspiration *via* connections to bulbospinal neurons in the rVRG (14). PiCo is an oscillatory neuronal population adjacent to the BötC and parafacial respiratory groups that is active in the post-inspiratory period (15). The pF_L_ drives expiration *via* connections to the bulbospinal neurons in the cVRG (1). Expiration is passive at rest and expiratory muscles are only recruited when metabolic/ventilatory demand is high, such as during exercise. The BötC contains inhibitory neurons that are active during post-inspiration and/or expiration and inhibit the preBötC and other brainstem regions (16). Inhibitory connections between the three oscillators are thought to maintain the three distinct phases of breathing (13). Forebrain regions with connections to the ventral respiratory column could potentially alter the inspiratory rhythm, coordination between the three oscillators/phases of breathing, or transmission of the respiratory rhythm to motor neurons.

### Parafacial respiratory group

The parafacial respiratory group consists of a lateral part (pF_L_) and a ventral part (pF_V_), located adjacent to the facial motor nucleus. The pF_L_ is thought to generate the rhythm responsible for the expiratory phase of breathing, as discussed above. The pF_V_ (also referred to as the retrotrapezoid nucleus) is critical for central chemosensation- responding to changes in pCO2 and pH in the blood (12). These two groups can be distinguished by their location as well as expression of Phox2b only in the pF_V_ (17, 18). Thus, forebrain connections to the pFRG region could potentially alter expiratory rhythm, control passive vs. active expiration, or modulate chemosensory responses.

### Dorsal respiratory group

The dorsal respiratory group consists of a population of respiratory neurons within the nucleus of the solitary tract (NTS) in the medulla. The NTS receives sensory input from the periphery and projects throughout the brainstem to modulate autonomic functions such as breathing and heart rate (19, 20). It is a major information processing and relay station, though only a portion of the NTS is involved in respiratory function. In the context of respiration, the NTS relays sensory afferent information from the vagal and glossopharyngeal nerves to the ventral respiratory column and receives information from the carotid bodies about circulating blood gas concentrations (19, 20). Therefore, forebrain regions connected to the dorsal respiratory group are likely to modify respiratory responses to peripheral sensory information or alter sensory processing.

### Pontine respiratory group

The pontine respiratory group is comprised of the Kölliker-Fuse nucleus and parabrachial nuclei. These are a collection of neurochemically diverse structures that are important for coordinating respiratory muscles during breathing and other orofacial behaviors such as vocalizing, coughing, swallowing, and emesis (21–23). The Kölliker-Fuse nucleus patterns respiratory muscle activity during the transition from the inspiratory to expiratory phases, mediates the inspiratory off-switch that triggers glottal closure, and controls upper airway patency (22, 24). The parabrachial nuclei pattern airway and expiratory muscle activity as well as receive chemosensory (i.e., hypoxia and hypercapnia) and mechanosensory information (i.e., negative airway pressure) important for arousal from hypercapnia (25, 26). These areas are likely critical for coordinating airway and respiratory muscles during diverse respiratory-related and orofacial behaviors driven by the forebrain.

### Periaqueductal gray

Located in the midbrain, the periaqueductal gray (PAG) serves as an interface between the forebrain and the brainstem to produce integrated behavioral responses to internal or external stressors (e.g., pain or threats) (27). The PAG coordinates respiratory, cardiovascular, and pain responses, as well as plays a part in vocalization, cough, sneeze, swallow, crying, laughter, micturition, arousal, thermoregulation, and sexual behaviors (27–30). As part of the “emotional motor system,” a major role of the PAG is likely to regulate breathing in response to emotional challenges and survival programs such as “fight or flight” or freezing responses (30).

The PAG receives inputs from the prefrontal cortex, amygdala, hypothalamus and nociceptive pathways and coordinates respiratory, cardiovascular, motor, and pain responses *via* efferents to the brainstem, forebrain and spinal cord (27, 30). Electrical or chemical stimulation of the PAG changes breathing patterns in rodents (31–33). Importantly, the pattern produced is dependent on which part of the PAG is stimulated. The effects of PAG stimulation on breathing include increasing respiratory frequency (e.g., tachypnea) and inspiratory effort (dorsal PAG, ventral part of lateral PAG), lengthening (e.g., apneusis) or shortening the inspiratory period (lateral PAG), or apnea (ventrolateral PAG) (32). The PAG is part of a descending system that modulates airway sensory processing, critical for control of breathing and breathing related behaviors, *via* projections to the nucleus of the solitary tract (29). The PAG can also directly control breathing through projections to the preBötzinger complex that modify the activity of pre-inspiratory neurons (32, 34). In addition, the PAG projects to the nucleus retroambiguus, a medullary region important for airway control during breathing, vocalization, and other behaviors (30, 35), as well as the Raphe nuclei and adrenergic nuclei (29). Thus, the PAG relays information from the forebrain to the brainstem to ensure that breathing patterns suit current behavioral or emotional needs such as fleeing predators, freezing, talking, crying, coughing, etc.

### Cerebellum

The cerebellum contains nearly half of the neurons in the central nervous system (36) and is a hub for processing information from many regions of the nervous system including the motor cortex, brainstem, and sensory afferents (37, 38). Although not principally a “respiratory region,” respiration is among the many motor and non-motor aspects modulated by the cerebellum (39–43). The ventral respiratory group sends numerous projections to the cerebellum and cerebellar deep nuclei send projections back to the ventral respiratory group (44–46), providing evidence that the cerebellum is actively involved in respiratory regulation. The cerebellum also has numerous reciprocal connections to other brainstem and forebrain regions, including those associated with responses to hypercapnia and air hunger (42–44). Additional evidence also suggests that the cerebellum is involved in the response to chemical (hypoxia and hypercapnia), and mechanical (tracheal occlusion and positive pressure breathing) respiratory challenges (41, 43, 45, 47). Although the role of the cerebellum in control of breathing is not fully understood, it's connectivity to both forebrain and brainstem respiratory centers makes it a potential hub for the forebrain to exert control over breathing.

## Forebrain control of breathing

The following sections will describe the anatomical and functional evidence supporting a role for each of the following structures in the control of breathing: cerebral cortex, amygdala, bed nucleus of the stria terminalis, hippocampus, hypothalamus, and thalamus (Figure 1).

### Cerebral cortex

The cerebral cortex of the brain is responsible for a diverse array of functions including voluntary motor functions, sensory processing, emotional processing, executive functions, attention, perception, memory, language, and cognition (48). Respiration is a motor function that is modified by sensory processes and emotional state, indicating that the cerebral cortex likely plays multiple roles in the control of breathing (49). This section will discuss evidence that different regions of the cerebral cortex likely play different roles in the control of breathing.

#### Motor cortex

The motor cortex is responsible for selecting, planning, and executing movements. Early evidence for a role of the cortex in breathing came in the late 1950's when it was noted that stimulation of the motor cortex in cats activated the phrenic nerve with a short latency, indicating a possible direct cortico-motoneuronal connection from the motor cortex to the phrenic motor nucleus (50), which was corroborated by various other studies (9, 51, 52). Investigators have suggested that activation of the diaphragm *via* the motor cortex is congruent with similar experiments that activate limb muscles through corticospinal pathways (53). In fact, the diaphragm region of the motor cortex can control forelimb muscles following a phrenic nerve transfer to the forelimb (54). It has since been shown that direct cortico-motoneuronal as well as cortico-reticulospinal and cortico-propriospinal-motor neuron pathways can mediate cortical control of breathing (9, 51, 55) and that these pathways do not involve medullary respiratory regions (9, 52, 56). The primary motor area, premotor area, and the supplementary motor area likely cooperate to modulate breathing. Respiratory linked activity has been observed in all three brain regions through a combination of EEG recordings, transcranial stimulation experiments, and neuroimaging efforts (57–61). Vocalizations require the deliberate and precise modification of respiration, and much of these signals originate in premotor cortices (11, 59, 62–64). The supplementary motor area exhibits a tonic drive to phrenic motoneurons that is thought to play an important role in the wakefulness drive to breathe as well as modulating breathing for speech (59, 63). A better understanding of how the motor cortex exerts volitional and tonic drive to spinal respiratory circuits could lead to new therapies to improve breathing in cases of disease or injury in which respiratory drive is insufficient.

#### Somatosensory cortex

The somatosensory cortex is activated by respiratory loads as well as low tidal volume, presumably *via* lung and chest wall mechanoreceptors (49). Low tidal volume also activates association motor cortices. Hypercapnia, which is sensed by carotid body and medullary chemoreceptors, does not activate the somatosensory cortex. Intriguingly, human subjects are able to distinguish between different magnitudes of respiratory loads, but not hypercapnia, consistent with the somatosensory cortex being important for discriminatory processes (49).

#### Insular and cingulate cortices

The insular and cingulate cortices have gained attention for their role in the sensation of dyspnea, the feeling of being unable to breathe (65–67). Therefore, these regions are particularly responsive to respiratory challenges such as hypercapnia, low tidal volume, and respiratory loads (49, 68–73). Due to their ties to the limbic system (65, 74) these regions also integrate emotional valence to sensory information (67, 71, 75) and likely play a role in generating behavioral responses to uncomfortable respiratory sensations (49).

### The extended amygdala: Amygdala and bed nucleus of the stria terminalis

The amygdala is part of the limbic system and evaluates the emotional importance of sensory information to prompt an appropriate response. It is known for its roles in processing fearful or threatening stimuli, reward processing, and stimulating aggressive behavior (5, 7, 76).

The bed nucleus of the stria terminalis (BNST) is a limbic structure adjacent to the amygdala that is also involved in fear and aggressive behaviors (7, 77, 78). We refer to these structures together as the extended amygdala due to their close functional association. This section of the review will discuss what is currently known about the role of the amygdala and the bed nucleus of the stria terminalis in the control of breathing and suggest directions for future research.

#### The extended amygdala has connections to brainstem respiratory regions

Functional imaging and electroencephalogram (EEG) studies have shown that the amygdala, along with other cortical and limbic brain regions, exhibits a high degree of coordination with the respiratory cycle predominantly within the lateral amygdaloid region (10, 76, 79). However, ablation of the amygdala has no effect on eupnea in rodents (80). Anterograde and retrograde tracing studies demonstrated that there are reciprocal connections between the extended amygdala and the ventral respiratory group (VRG) (44), which could potentially mediate the coordination of activity. In fact, the central nucleus of the amygdala (the output center of the amygdala), has direct, monosynaptic projections to the preBötzinger Complex, as identified by viral tracing experiments (11, 81). Although the central nucleus is predominantly composed of inhibitory GABAergic neurons, it has connections to both excitatory and inhibitory neurons of the preBötzinger Complex (preBötC) and thus is poised to exert a variety of effects on the preBötC, possibly dependent upon emotional states (82–85). These reciprocal connectivity between the extended amygdala and VRG may serve as a substrate to regulate breathing in response to fear/anxiety, regulate fear-related behaviors in response to breathing rhythm, or both. Functional testing of this hypothesis in animal models awaits future studies.

The extended amygdala also has connections to other brain regions important for the control of breathing. Retrograde-tracing studies in rats and mice have shown that both the bed nucleus of the stria terminalis and central nucleus of the amygdala have afferent projections to the nucleus of the solitary tract, which is the viscerosensory tract that bears information from the vagus nerve among others (19, 86). However, since both the nucleus of the solitary tract and extended amygdala have broad functions in autonomic control, it is unclear whether this connection has a role in respiration. The midline apneic site, a medullary brain region related to the raphe nuclei and partially containing the raphe pallidus, receives projections from both the BNST and central nucleus of the amygdala (87). This connection could provide an explanation for the respiratory depression and central apnea that is elicited by stimulation of the central amygdala and bed nucleus of the stria terminalis (see below).

#### Amygdala stimulation can inhibit breathing

Direct proof that the amygdala can influence breathing comes from animal studies demonstrating that electrical stimulation of the lateral amygdala region can reduce phrenic nerve output and slow ventilation (82). Working with human epilepsy and non-epileptic patients, multiple studies have noted that electrical stimulation of the amygdala can cause apnea (83, 85, 88, 89). The location of this site, called the amygdala inhibition of respiration (AIR) site, has been mapped in pediatric patients (85). Intriguingly, stimulation of the amygdala in excess of 30 s evoked apnea; yet the subjects showed no signs of discomfort (i.e., dyspnea) or arousal (83, 85). For comparison, subjects were unable to voluntarily hold their breath for longer than 20 s without experiencing dyspnea. Interestingly, patients stimulated in the amygdala were still able to breathe by bringing their attention (6, 89). Another interesting finding is that apnea only occurs when the patient is breathing through their nose; apnea did not occur following amygdala stimulation when the patient was instructed to breathe through their mouth (89). This discovery is likely mediated through higher brain regions, as voluntary breathing is known to override amygdala stimulation-induced apnea and cortical structures have a known impact on volitional breathing. These findings may have important implications for sudden death in epilepsy (SUDEP) or sudden infant death syndrome (SIDS) as they suggest a mechanism whereby altered amygdala function could cause prolonged apneas while at the same time inhibiting dyspnea and arousal, leading to death.

#### The extended amygdala and sudden death in epilepsy

There is a growing body of evidence implicating the extended amygdala in seizure-induced respiratory dysfunction. In several mouse models of epilepsy, induction of seizures can result in central apnea and peri-ictal respiratory depression that can lead to death (80, 90–92). Studies in epileptic patients have shown that seizure spread to the amygdala is correlated with apneas (83, 85, 93). Moreover, electrolytic lesions of the amygdala reduce the occurrence of seizure-induced respiratory arrest in a mouse SUDEP model (80). Together with the studies described above showing that direct stimulation of the amygdala can cause apneas (see: *Amygdala stimulation can inhibit breathing*), these findings suggest that aberrant activity of the amygdala during or following seizures could lead to breathing dysfunction and SUDEP. Currently, it is not clear whether the amygdala directly alters breathing *via* connections to the VRG, NTS and other brainstem structures, and/or alternatively *via* other forebrain or midbrain regions. The bed nucleus of the stria terminalis has also been implicated in seizure-induced respiratory changes as these neurons are activated by seizures in a mouse model of Dravet syndrome (78). The investigators demonstrated that neurons projecting from the bed nucleus of the stria terminalis to the parabrachial nucleus (a pontine structure that regulates breathing) are hypoexcitable in Dravet syndrome mice, suggesting a potential circuit leading to breathing dysfunction and SUDEP (78). Additional studies are warranted to identify and further test which circuits are responsible for seizure-induced breathing abnormalities in different forms or models of epilepsy and how they might lead to SUDEP.

### Hippocampus

The hippocampus is a structure located in the archicortex or allocortex and is considered an extension of the temporal lobe of the cerebral cortex although anatomically and functionally distinct (94). Main functions of the hippocampus include, but are not limited to, emotional processing, memory and learning, and spatial navigation. The role that the hippocampus plays in respiration is still not well understood but current evidence will be discussed below.

#### Lack of evidence for direct connectivity to brainstem respiratory centers

Viral tracing studies in rats demonstrate that the hippocampus receives connections from the nucleus of the solitary tract *via* polysynaptic pathways, suggesting the hippocampus may play a role in processing afferent information from the vagus nerve (95). However, whether the hippocampus receives information from lung afferents has not yet been determined. Diffusion imaging studies in humans provide evidence that there are connections between the hippocampus and the brainstem and cervical spinal cord (96). Electrophysiological studies in rodents have demonstrated sleep-state dependent functional connectivity between the hippocampus and brainstem (97). However, the imaging and electrophysiological studies could not assess the direction of signaling between the brainstem and hippocampus. Moreover, evidence of direct connections from the hippocampus to brainstem respiratory centers is lacking (44, 76, 81, 87, 98). Effects of the hippocampus on breathing are likely to be indirect (i.e., polysynaptic), mediated through another region such as the thalamus, cortex or amygdala, or the connections could be so sparse that they are difficult to label and trace.

#### Hippocampus stimulation alters breathing

Cells of the hippocampus have also been reported to discharge in phase with respiratory patterns in humans and rodents (10, 84, 99–102). Electrical stimulation of the hippocampus can result in the cessation of breathing (6, 88, 103). Like the amygdala, hippocampal-produced apnea can be overcome by volitional breathing or speech (6). Varying the levels of stimulation intensity or location can result in a spectrum of breathing differences (6). For example, stimulating the hippocampus during expiration can halt expiration or induce a phase switch to inspiration (100). The hippocampus may also play a role in triggering sighs, or “augmented breaths.” Sighs are a normal component of breathing driven by the preBötzinger complex that are important for reinflating collapsed alveoli (1), but are also associated with the expression of mood and emotions. Poe et al. (101) noted that hippocampal activity in freely behaving cats increased prior to the initiation of a sigh or the end of an apnea. However, another study demonstrated that electrical stimulation of the ventral hippocampus inhibits sighs (99). Thus, it has been proposed that the hippocampus may play a role in controlling the timing of sighs (99). The ventral region of the hippocampus is a primary region dealing with fear and anxiety. Thus, it seems likely that the hippocampus is part of a limbic circuit that controls emotional aspects of sighing *via* indirect connections to the preBötzinger complex.

### Hypothalamus

The hypothalamus is a structure located just above the brainstem with well-known roles in homeostatic regulation: from metabolism and endocrine function to sleep regulation and the circadian rhythm (48, 104). Since the hypothalamus is involved in many autonomic processes, researchers have explored its role in respiration. For a more focused discussion on the role of the hypothalamus in control of breathing, we recommend Fukushi et al. (105). Here, we first discuss the main neuropeptide hormones produced by the hypothalamus that influence breathing (orexin and vasopressin). We then describe potential roles of the distinct hypothalamic regions in the control of breathing, including: the paraventricular nucleus, perifornical area, dorsomedial region of the hypothalamus, and lateral and posterior hypothalamus.

#### Neuropeptide hormone signaling by the hypothalamus

The hypothalamus is responsible for the production and release of numerous hormones that regulate a broad variety of autonomic and behavioral functions (106–108). Two in particular, vasopressin and orexin, appear to be involved in respiratory physiology and will be discussed below (109, 110).

Vasopressin, also known as antidiuretic hormone, is generated in the paraventricular and supraoptic nuclei of the hypothalamus by processing of the same pre-pro-hormone that generates neurophysin II and copeptin (109). It is well known for its role in maintaining the balance of water and electrolytes in the kidneys and circulatory system but can also influence other homeostatic functions such as glucose regulation, cardiovascular regulation, and breathing (109). Vasopressin can act as a hormone in the periphery or as a neuropeptide within the central nervous system by binding to one of three different G-protein coupled receptors (V1a, V1b, V2) (109). The receptor most significant for the control of breathing is likely the V1a receptor (V1aR), which is expressed in the lungs, carotid bodies, and circumventricular organs (subfornical organ, area postrema, and organum vasculosum laminae terminalis). Within the brainstem, V1aRs can be found in the rostral ventrolateral medulla, the rostral ventral respiratory column, and the preBötzinger complex, as well as the nucleus of the solitary tract, and the phrenic nuclei (111–113). A link between vasopressin and control of breathing was established by studies showing that the same stimuli that release vasopressin also result in changes to ventilation (114–118). The effects of circulating or central release of vasopressin on breathing can vary by target region, but it is generally inhibitory to breathing (109). Vasopressin is released during physical exercise, and it has been noted that increased levels of vasopressin accompany respiratory disorders such as COPD and pneumonia (119). Moreover, the expression of V1aRs has been shown to change in response to respiratory stresses. For example, hypoxia has been shown to increase the expression of V1aRs in the rostral ventrolateral medulla, the ventral respiratory column, and the phrenic nuclei (113). Additionally, hypercapnia has been shown to activate the vasopressinergic neurons of the paraventricular nucleus in the hypothalamus (120). Thus, vasopressin likely plays a homeostatic role in the control of breathing by modulating the function of multiple brain and spinal cord regions in response to activity or respiratory stress. Although it is not clear why a hormone that is generally inhibitory to breathing is released under conditions of respiratory stress, it has been proposed to play a protective role in preventing hyperventilation (109). Additional research is warranted to better understand the role of vasopressin in control of breathing.

Orexin (also known as hypocretin) is a neuropeptide expressed exclusively in the hypothalamus that acts on G-protein coupled receptors throughout the central nervous system (110, 121, 122). It has two forms (orexin-A and orexin-B) derived from the same precursor protein. The loss of orexinergic neurons leading to narcolepsy, demonstrating its critical role in promoting wakefulness (123, 124). Orexin has also been implicated in regulating aspects of metabolism, homeostasis, reward seeking behavior, and respiration (122, 125). Orexinergic neurons can be found in the perifornical area, dorsomedial hypothalamus, and lateral hypothalamus (110, 121, 126) and have known projections to respiratory regions, including: the preBötzinger complex, nucleus of the solitary tract, Kölliker-Fuse nucleus, parabrachial nuclei, and the phrenic nucleus of the spinal cord (122, 124, 127–132). Orexin neurons are sensitive to CO2, implicating them in chemosensory responses (133, 134). Orexin is likely important for sleep state-dependent regulation of breathing, as its expression is greatest during wakefulness. Further, mice lacking orexin show a 50% decrease in the respiratory response to CO2 during wakefulness, but not during sleep (135), which can be remedied by orexin supplementation (123). Consistent with this data, orexin deficiency can lead to sleep apneas in animal models (123) and decreased orexin levels are found in patients with obstructive sleep apneas (136). At least some of the stimulatory effects of orexin on breathing appear to be mediated through projections to the Kölliker-Fuse nucleus, as injection of orexin-B into this region increases the respiratory frequency in rodent brainstem preparation (137). A better understanding of the role of orexin in sleep state-dependent regulation of breathing and arousal could have important implications for sudden infant death syndrome (SIDS) or sudden death in epilepsy (SUDEP).

#### Paraventricular nucleus

The paraventricular nucleus (PVN) is predominantly known for its role in the regulation of various autonomic functions including stress responses, metabolism, and reproduction (138–140). The PVN has vast connections to brainstem regions important for respiratory control including the periaqueductal gray, parabrachial nucleus, retrotrapezoid nucleus, nucleus of the solitary tract, preBötzinger complex, and the phrenic nucleus in the spinal cord (105, 139, 141, 142). Experiments in rats and rabbits found that electrical or chemical (glutamate) stimulation of the paraventricular nucleus increases respiration (140, 143). The paraventricular nucleus receives afferent input from other parts of the hypothalamus, as well as the subfornical organ (a chemosensory organ), and the BNST (see: *The extended amygdala: amygdala and bed nucleus of the stria terminalis*) (144). Inputs from the hippocampus, amygdala, and lateral septum can influence magnocellular neurosecretory cells in the PVN, likely *via* short projections from other parts of the hypothalamus and/or from the BNST. Thus, the PVN may serve as a relay station or integration center for other forebrain regions to influence the brainstem and/or spinal circuits controlling breathing.

#### Perifornical area

The perifornical area is commonly known for its role in the hypothalamic defense system, which is important for the assessment of threats, and predatory threats in particular (105, 126, 145, 146). The perifornical area is a widely interconnected region, showing projections to the nucleus of the solitary tract, Kölliker-Fuse nucleus, parabrachial nuclei, and periaqueductal gray (121, 123, 147–153). Chemical inhibition of the perifornical area abolishes the respiratory response to stressful auditory and visual stimuli in rats (145). Further, chemical disinhibition of the perifornical area of rats increases respiration (146). Thus, this area is likely to work with limbic structures to drive appropriate respiratory responses to stress and fear.

#### Dorsomedial hypothalamus

The dorsomedial hypothalamus plays a prominent role in response to stress and arousal (105, 154) and is crucial for the processing of respiratory and other autonomic changes in response to psychological stressors (145, 155). Congruent with its role in stress and arousal, the dorsomedial hypothalamus receives dense projections from the amygdala and bed nucleus of the stria terminalis (126, 156). The dorsomedial hypothalamus is known to send projections to the ventral respiratory column, as well as the periaqueductal gray, nucleus of the solitary tract, and Kölliker-Fuse nucleus/parabrachial nuclei (121, 123, 149, 152, 157–160). Disinhibition of the dorsomedial hypothalamus by injecting bicuculline in rats leads to increased respiratory drive and hyperventilation (161, 162). Working in concert with the perifornical area, these neighboring regions form the center of the hypothalamic defense area that is known to elicit a variety of sympathetic changes including increases in cardiovascular and respiratory activity in response to stress (126, 146, 163).

#### Lateral hypothalamus

The lateral hypothalamus is classically known as the “feeding center” because of its association with driving the motivation to eat and drink (164, 165) and also plays a role in controlling sleep/wake states (122, 123). Destruction of the lateral hypothalamus or inhibition by barbiturates has been shown to decrease the frequency and depth of ventilation (104). The lateral hypothalamus contains orexinergic neurons and has known projections to the rostral ventral respiratory group (44). The lateral hypothalamus also has a role in central chemosensation as neurons in this region respond to changes in the levels of carbon dioxide (133, 134). This region is also known to receive projections from the preBötzinger Complex (166). Chemosensory activation of the lateral hypothalamus is likely to regulate breathing at least in part *via* release of orexin, which is released maximally during wakefulness (134) (see: *Neuropeptide hormone signaling by the hypothalamus*). This region is thus likely important for sleep/wake state-dependent regulation of breathing.

#### Posterior hypothalamus

The posterior hypothalamus, also referred to as the caudal hypothalamus, is involved in a variety of behaviors and processes including: cardiovascular regulation, cardiorespiratory responses, locomotion, circadian rhythms, and defense responses (105, 106, 110, 167). This region has strong connections to the periaqueductal gray and medullary respiratory centers (168, 169). The posterior hypothalamus may play a role in the respiratory increase that accompanies movement (i.e., exercise hyperpnea) (105, 155, 170). Prior to and during exercise, both feedforward (central command) and feedback (chemosensory) signaling mechanisms ensure that respiration is able to provide enough oxygen for the increase in metabolic demand. The posterior hypothalamus contains chemosensitive neurons that respond to hypoxia and facilitate respiration as well as GABAergic neurons that modulate the respiratory response to hypercapnia (105, 171, 172). The posterior hypothalamus likely plays a role in the feedforward mechanism of exercise hyperpnea as electrical stimulation or chemical disinhibition are able to induce both increased respiration and spontaneous locomotion (170, 173, 174). These results indicate that the posterior hypothalamus uses multiple mechanisms to ensure that respiratory activity is matched to behavioral and metabolic demands.

### Thalamus

The thalamus is a structure of the diencephalon important for relaying sensory information to the cortex and motor information from the cortex to other brain regions (175–178). There are several thalamic nuclei that are known to send direct, monosynaptic projections to the rVRG, namely the parafascicular, mediodorsal, and subparafascicular nuclei (44). The thalamus may also influence respiration indirectly *via* connections to the cerebral cortex, hippocampus, extended amygdala or other brain areas that control breathing.

As different parts of the thalamus play different roles in gating sensory and motor information, it is not surprising that different parts of the thalamus appear to have different effects on breathing. Electrical stimulation of the mediodorsal nucleus of the thalamus in cats can increase respiratory rate (179). However, electrical stimulation of the parafascicular nucleus of the thalamus in fetal sheep reduces respiratory frequency (180). Consistent with this finding, lesions to the posteromedial thalamus, and particularly the parafascicular nuclei, abolished the normal response of the fetus to hypoxic conditions (181, 182). This region of the thalamus is involved with sleep regulation postnatally (181), but its involvement in sleep-disordered breathing is currently unclear. In a rat model for obstructive sleep apnea, rats exposed to chronic intermittent hypoxia revealed increased *c-fos* expression in the paraventricular thalamus (183). In this situation, the increase in neuronal activity indicated by the increase in *c-fos* expression suggests that the thalamus plays an important role in the stress response as it relays information to the prefrontal and insular cortices. Additional research is needed to better understand how sensory (or chemosensory) information is gated by the thalamus as well as how respiratory motor output may be processed for the control of breathing.

## Conclusions

**Figure 2:**
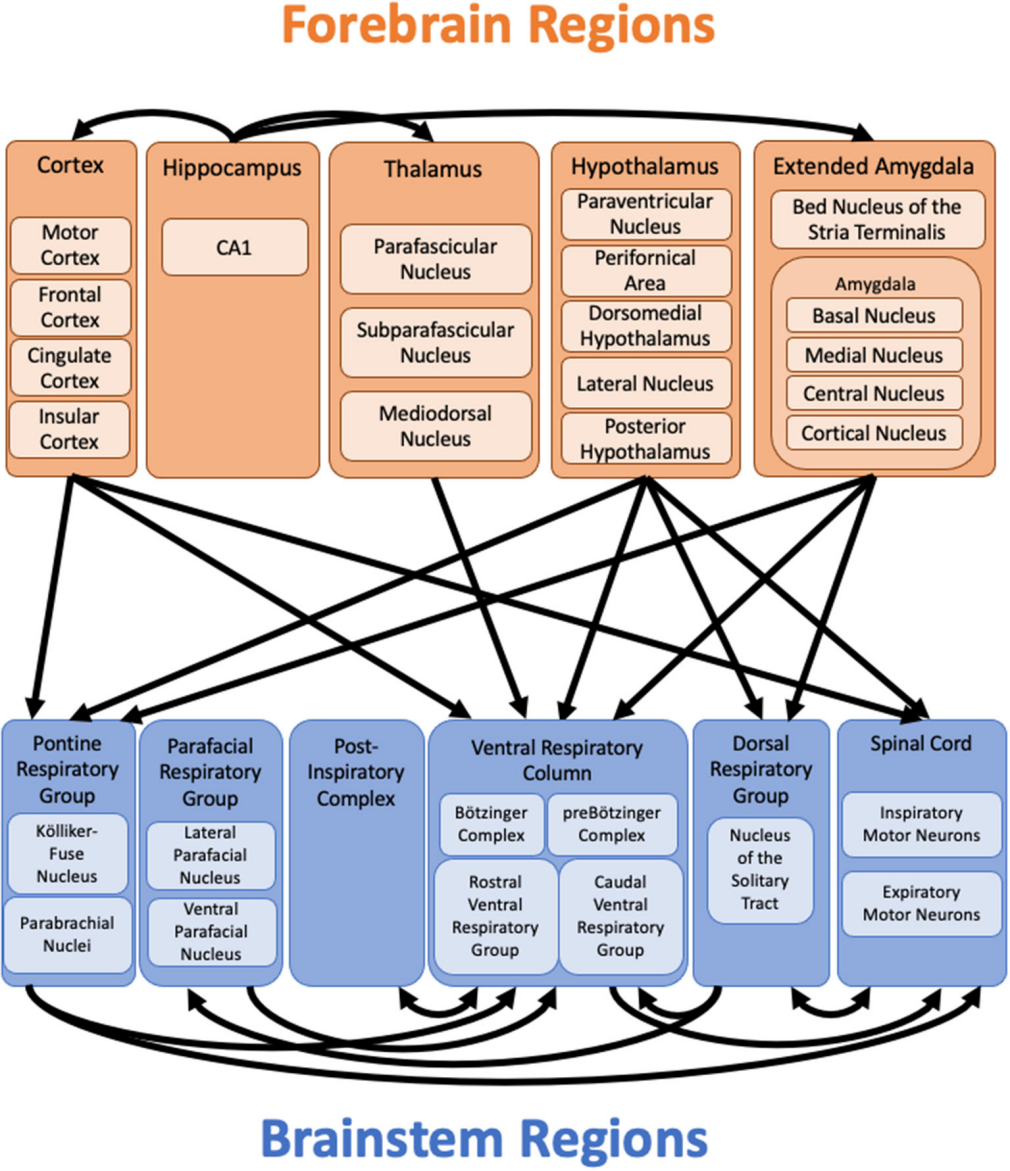
Forebrain projections to brainstem respiratory regions. Diagram of known projections from forebrain regions to brainstem respiratory regions as discussed in the text. Connections from the brainstem to forebrain regions are not shown. Connections from the hippocampus to other forebrain regions highlight potential pathways through which the hippocampus might influence respiration in lieu of known direct projections to brainstem respiratory regions. The extensive connections between other forebrain regions are not shown. Important connections within the brainstem highlight additional pathways through which the forebrain might influence breathing, even in the absence of direct connections to specific brainstem respiratory regions. Additional regions, such as the cerebellum and periaqueductal gray (not shown), may relay information from the forebrain to the brainstem.

Our brain uses a variety of feedback, feedforward, and homeostatic mechanisms to ensure that our breathing is appropriately matched to our physiological, emotional, and behavioral state. We propose that the forebrain regions reviewed here (the cerebral cortex, extended amygdala, hippocampus, hypothalamus, and thalamus) contribute to the regulation of breathing for this purpose. These forebrain regions have multiple connections to each other, as well as direct or indirect connections to brainstem regions known to be important for the control of breathing (Figure 2). For each region, there is functional evidence that they play a role in the control of breathing, at least during certain behaviors, conditions, or physiological states. Further research is necessary to elucidate the roles of these forebrain structures in the control of breathing under different conditions as well as the specific circuits and mechanisms involved.

## Author contributions

KS and SC conceived, designed, and wrote the manuscript. KS designed and generated the figures. SC edited the figures and manuscript. All authors contributed to the article and approved the submitted version.

## Funding

Our research on forebrain control of breathing is supported by the Citizens United for Research in Epilepsy (CURE) Foundation and NIH R21 NS121644-01.

## Conflict of interest

The authors declare that the research was conducted in the absence of any commercial or financial relationships that could be construed as a potential conflict of interest.

## Publisher's note

All claims expressed in this article are solely those of the authors and do not necessarily represent those of their affiliated organizations, or those of the publisher, the editors and the reviewers. Any product that may be evaluated in this article, or claim that may be made by its manufacturer, is not guaranteed or endorsed by the publisher.
